# The Immune System's Contribution to the Clinical Efficacy of EGFR Antagonist Treatment

**DOI:** 10.3389/fphar.2017.00575

**Published:** 2017-08-24

**Authors:** Felicity MacDonald, Dietmar M. W. Zaiss

**Affiliations:** School of Biological Sciences, Institute of Immunology and Infection Research, University of Edinburgh Edinburgh, United Kingdom

**Keywords:** EGFR antagonists, regulatory T cells, immunotherapy of cancer, immune responses, efficacy

## Abstract

Epidermal Growth Factor Receptor (EGFR) antagonists were one of the first anti-cancer treatments developed targeting a Receptor Tyrosine Kinase. However, the underlying mode of action of how EGFR antagonist application can explain its clinical efficacy in different types of cancers remains largely unresolved. Numerous findings have suggested that a substantial portion of the effects attributed to EGFR antagonist treatment might not be based on *direct* influence on the tumor itself. Instead it may be based on *indirect* effects, potentially mediated via the immune system. In this review the role of the EGFR for the functioning of the immune system is discussed, alongside how EGFR antagonist treatment could be impacting tumor growth by blocking macrophage and FoxP3-expressing regulatory CD4+ T cell function. Based on these findings, we consider implications for current treatment schemes and suggest novel approaches to improve the efficacy of EGFR antagonist treatment in the future. Finally, we propose potential ways to improve EGFR antagonists, in order to enhance their clinical efficacy whilst diminishing unwanted side effects.

The Epidermal Growth Factor Receptor (EGFR) was the first Receptor Tyrosine Kinase to be described (Gschwind et al., 2004). Due to the fact that many tumors of epidermal origin express high levels of this cell surface receptor, antagonists targeting the EGFR were also amongst the first biologicals approved for the treatment of cancer patients (Ciardiello and Tortora, 2008). Examples of such antagonists include two EGFR blocking antibodies, Cetuximab and Panitumumab, as well as two chemical tyrosine kinase inhibitors, Erlotinib and Gefitinib. Many further antagonists are now in advanced development. These EGFR antagonists show considerable clinical efficacy and, in particular, their use in colon carcinoma as well as that in head and neck cancer treatment can substantially extend survival time (Ciardiello and Tortora, 2008).

Fundamentally, the clinical efficacy of EGFR antagonists in cancer treatment was an unexpected finding, as the EGFR is ubiquitously expressed throughout the human body and not itself an oncogene. Contrary, deletion of the EGFR specifically in hepatocytes has been shown to lead to enhanced development of liver cancer in mice (Lanaya et al., 2014), demonstrating that this receptor may have beneficial properties in the protection against cancer. Only a mutated form of the EGFR, known as EGFRvIII, can cause cancer. This mutation deletes exons 2 to 7 of the EGFR gene, leading to low-level constitutive signaling that can drive tumor progression (Gan et al., 2013).

A number of different direct modes of action have been suggested that may explain the clinical efficacy of EGFR antagonist treatment in cancer therapy. One of the original suggestions was that tumors grow “addicted” to growth factor signaling. As such, interruption of this signaling was assumed to lead to cancer cell death. However, this suggestion was substantially based around the fact that many tumors strongly overexpress the EGFR, and it was assumed that such an extreme overexpression of this receptor would supposedly lead to higher sensitivity of the tumor cells to growth factor-induced proliferation. Nevertheless, it was soon recognized that tumor-specific overexpression of the target molecule was not a prognostic marker of tumor treatment (Burtness et al., 2005; Kim et al., 2008), as tumors that did not express detectable levels of the EGFR still reacted to antagonist treatment such as monoclonal antibody therapy (Chung et al., 2005). It has also been shown that tumors responsive to EGFR antagonist treatment *in vivo* are often not sensitive to monoclonal antibody treatment in cell culture when explanted (López-Albaitero and Ferris, 2007).

As alternative modes of action, in particular with antibody-based treatments, complement-mediated, and natural killer (NK) cell-mediated killing of tumor cells has been suggested. However, as the EGFR is also ubiquitously expressed on healthy tissues, such modes of action would only be able to explain for the clinical efficacy of treatment on tumor cells that express an elevated level of the target molecule. Thus, it appears reasonable to assume that the clinical efficacy of EGFR antagonist treatment may not be based on *direct* effects on the tumor, but may also be in part based on *indirect* effects. One such possibility may be the interaction of antagonists with EGFR expression on healthy cells in the tumor microenvironment, such as tumor-intrinsic fibroblasts, or on cells of the immune system. In support of such an assumption it has been reported that Cetuximab treatment in particular can activate the host anti-tumor immune response (López-Albaitero and Ferris, 2007; Yang et al., 2013). Furthermore, Garrido et al. demonstrated, in a mouse model of lung carcinoma, that the anti-metastatic effect of EGFR inhibitor treatment crucially depends upon the immune system (Garrido et al., 2007). Depletion of either CD8+ or CD4+ T cells abrogated the beneficial effects seen following EGFR inhibitor treatment (Garrido et al., 2007), therefore these findings strongly suggest that the immune system may substantially contribute to the clinical efficacy of EGFR antagonist treatment.

This review will discuss evidence that implicates the involvement of the immune system in EGFR antagonist-based tumor treatment, considering the measures required to improve current treatment in order to enhance clinical efficacy and diminish any associated side effects.

## Role of the EGFR in the immune system

It has been well-established that the EGFR is expressed on many different haematopoietic cell types and that its expression is of central importance for their functioning. These cell types include macrophages (Scholes, 2001; Lanaya et al., 2014), monocytes (Chan et al., 2009), plasma cells (Mahtouk et al., 2005), and certain T cell subsets such as effector CD4 T cells and FoxP3-expressing regulatory CD4 T cells (Tregs) (Zaiss et al., 2013). It is therefore plausible that EGFR antagonists used to target tumors can interfere with the functioning of the immune system. This potentially explains for the enhanced susceptibility to infections seen in patients treated with these antagonists (Burtness et al., 2012), and the observed mortality of patients arising from severe bacterial infections when treated with immunosuppressant mTOR inhibitors in combination with EGFR inhibitors (Burtness et al., 2012).

Tumors require interaction with many different host immune cell populations for their growth and survival. Mast cells, for instance, are recruited to tumor environments where they mature and release angiogenic mediators to support the development of new blood vessels and provide growth factors to support tumor development (Maltby et al., 2009). Tumor associated macrophages (TAM) are another key immune cell type implicated in tumor growth. TAMs have been found to stimulate angiogenesis, as well as secreting molecules that enhance tumor cell proliferation and metastasis, and promoting tumor progression by establishment of a suppressive microenvironment (Liu and Cao, 2015). Additionally, other suppressive cell types are also found in the tumor microenvironment, including regulatory T cells (Treg) and myeloid-derived suppressor cells (MDSC). These cells dampen the anti-tumor immune response by interacting with cells such as NK cells, T cells, and dendritic cells (DC) (Zou, 2006; Ostrand-Rosenberg and Sinha, 2009). Thus, it is reasonable to assume that EGFR antagonist interference with any of these leukocytes' function may advantageously contribute to the clinical efficacy of anti-tumor treatments.

## Role of EGFR expression for macrophage function

Macrophages make up a substantial component of many tumors. Clinical studies have indicated that cancers containing high infiltrates of macrophages are associated with poor prognosis for the patient (Zhang et al., 2012). Recent studies in a number of different mouse tumor models have revealed that EGFR-mediated signaling, specifically within macrophages, substantially contributes to the initiation of tumor growth (Lanaya et al., 2014; Hardbower et al., 2017; Srivatsa et al., 2017). In line with these findings, the group of John Condeelis revealed an EGFR based cross-talk between macrophages and tumor cells (Wyckoff et al., 2004). In a paracrine loop intra-tumoral macrophages secrete EGF, which binds to the EGFR on tumor cells, promoting their invasion. At the same time, tumor cells secrete colony-stimulating factor-1 (CSF-1) which in turn promote the expression of EGF by macrophages (Wyckoff et al., 2004). Nevertheless, in all of the mouse tumor models used, EGFR gene-deletion in the cancerous target tissue had no influence on the development of tumors. However, EGFR gene-deletion in macrophages did influence tumor development. This suggests a role for macrophage-intrinsic EGFR-mediated signaling in the establishment of the tumor microenvironment. Mechanistically it has been shown that EGFR expression regulates macrophage cytokine production (Lanaya et al., 2014; Hardbower et al., 2016), and that macrophage-derived cytokines are predominant drivers of tumorigenesis (Lanaya et al., 2014; Hardbower et al., 2017; Srivatsa et al., 2017). Thus, by blocking EGFR function in tumor residential macrophages during EGFR antagonist treatment this supply of cytokines may be interrupted, leading to diminished tumor growth and tumor-intrinsic instability.

## Role of EGFR expression for regulatory T cell function

One further immune cell population of particular interest in cancer treatment are FoxP3-expressing regulatory T cells (Tregs). The role of EGFR expression for Treg functioning is also well-established (Zaiss et al., 2013) and Tregs play a role in maintaining immune homeostasis by negatively regulating other immune cell types. Thereby Tregs prevent the development of autoimmunity through establishment of peripheral tolerance to self-antigens. Tumors are able to exploit the suppressive nature of Tregs found within the tumor microenvironment, and Tregs have been shown to traffic to, proliferate and mature within the tumor microenvironment under influence of local factors produced by tumors and associated cells (Zou, 2006). As such, the presence of Tregs allows tumors to escape immune surveillance as they dampen anti-tumor immune responses, enabling the induction of immunological tolerance against tumor-antigens.

For optimal functioning, both human and murine Tregs are dependent on EGFR-mediated intrinsic signaling upon binding of the ligand Amphiregulin (AREG) (Zaiss et al., 2013). AREG is known to be a type II cytokine involved in wound healing and contributes to host resistance to helminth infections (Zaiss et al., 2006, 2015). In contrast to most other EGFR ligands, the binding of AREG to the EGFR induces a prolonged, tonic signal through the MAP-kinase (MAPK) signaling pathway that does not lead to the internalization of the receptor. Tregs express the EGFR under inflammatory conditions, as witnessed by elevated levels of EGFR found on tumor-infiltrating Tregs derived from wild type (wt) mice with B16 melanomas, as well as EGFR expression on human Tregs with an activated phenotype of FoxP3^hi^ and CD45RA- (Zaiss et al., 2013). *In vitro* treatment of EGFR-expressing Tregs with AREG enhances their suppressive capacity, shown by decreased proliferation of peripheral blood mononuclear cells (PBMC) incubated with both Tregs and AREG. The group of Mark Wilson further demonstrated that the addition of AREG increases the release rate of microRNA-containing exosomes from Tregs, which these cells use as a means of Treg-mediated immune suppression (Okoye et al., 2014). In consequence, Tregs that either lacked EGFR expression or were transferred into an AREG-deficient environment could not suppress the development of autoimmune diseases in both a dermatitis and a T-cell transfer-based model of colitis (Zaiss et al., 2013). This clearly demonstrates that Tregs are directly dependent on AREG-induced and EGFR-mediated signaling for their *in vivo* functioning.

It is widely accepted that Tregs play a pivotal role in creating the suppressive tumor microenvironment. Using a well-established murine B16 melanoma immunization model (Sutmuller et al., 2001), it has been demonstrated that AREG plays a critical role in such Treg-mediated tumor immune suppression (Zaiss et al., 2013). In B16 tumors, Tregs protect against immunization-induced CD8+ T cell-mediated anti-tumor immune responses. It has been shown that when C57BL/6 wt mice were transplanted with B16 tumors, and an anti-tumor CD8+ T cell response induced by immunization with immunogenic tumor-epitope pulsed bone marrow-derived dendritic cells (BM-DC), large tumors grew 3 weeks following tumor transfer. In contrast, BM-DC immunized *Areg* gene-deficient mice and mice lacking EGFR expression specifically on Tregs (*FoxP3-cre x EGFR*^*fl*/*fl*^) (unpublished data) efficiently rejected the transplanted tumors upon immunization (Zaiss et al., 2013). These findings demonstrate that AREG enables Tregs to suppress anti-tumor immune responses.

Remarkably, mast cells have been determined to be the critical source of AREG, enhancing Treg function (Zaiss et al., 2013). It has been shown before that mast cells can cooperate with Tregs to suppress skin transplant-specific immune responses, a model in which the immunosuppressive function of Tregs is well-established to induce immunological tolerance (Lu et al., 2006). Furthermore, in a model of T cell transfer-induced colitis, using a mast cell-deficient mouse strain *c-kit*^*w-sh*/*w-sh*^ backcrossed onto a *RAG1*^−/−^ background, it was demonstrated that mice reconstituted with *wt* bone marrow-derived mast cells (BM-MC) prior to co-transfer of naïve CD4+ T cells and Tregs had a much lower colitis score than mast cell-deficient mice that had either not been reconstituted or reconstituted with *Areg*^−/−^ BM-MC. This clearly demonstrates that mast cell-derived AREG is essential to restore the suppressive capacity of transferred Tregs (Zaiss et al., 2013).

**Figure 1:**
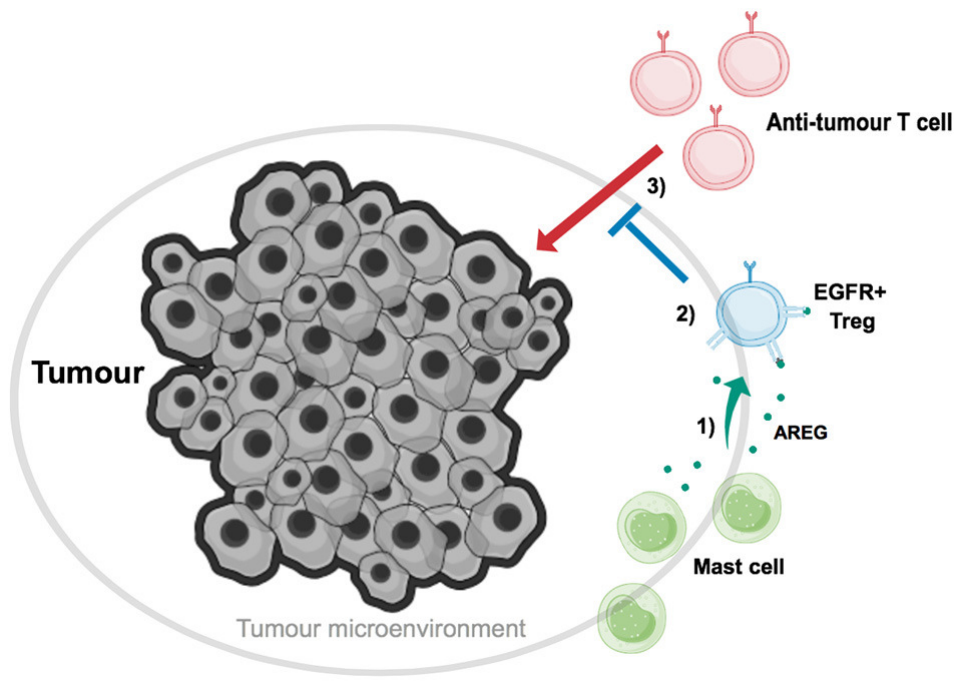
Mast cell-derived Amphiregulin enables tumor-residential regulatory T cells to suppress anti-tumor immune responses. Tumor-associated Tregs (blue) have been found to localize on tumor margins, along with mast cells (green). **(1)** Mast cells cooperate with Tregs, and aid in tumor growth, through secretion of the EGFR-ligand AREG (dark green). **(2)** AREG binds to EGFR on Tregs, inducing intrinsic signaling which ultimately enhances Treg suppressive function. **(3)** Tregs, with enhanced EGFR-mediated suppressive abilities, can now suppress the action of anti-tumor T cells, and thus aid in tumor immune evasion.

In further accordance with these findings, it has also been demonstrated that the efficacy of tumor immune therapy is enhanced in mast cell deficient mouse strains (Wasiuk et al., 2012; Zaiss et al., 2013). Mast cells are found to accumulate at the edge of tumors, and in some tumor types this accumulation has been shown to correlate with a poor prognosis for cancer patients (Takanami et al., 2000). Tumor-associated Tregs have also been shown to localize on tumor margins, within so-called tertiary lymphoid structures (TLS) (Joshi et al., 2015). TLSs are often formed at sites of infection, inflammation and cancer, and similarly to secondary lymphoid structures they contain B cell and T cell zones along with high endothelial venules. This composition allows for the formation of efficient adaptive immune responses, as TLSs allow entry of immune cells into the tumor environment and priming of lymphocytes. It is here that Tregs are believed to suppress anti-tumor immune responses. Reconstitution of mast cell-deficient *c-kit*^*w-sh*/*w-sh*^ mice with *wt* BM-MC prior to tumor transfer, and immunization with tumor-epitope pulsed BM-DCs, led to enhanced resistance to tumor immune therapy. However, when *c-kit*^*w-sh*/*w-sh*^ mice were reconstituted with *Areg*^−/−^ BM-MC prior to immunization, tumor protection against the induced immune responses was lost. These findings highlight that mast cell-derived AREG increases the suppression of Tregs *in vivo*, thereby enabling tumor-resident Tregs to suppress anti-tumor immune responses (Figure 1).

Taken together, targeted interference with Treg-intrinsic EGFR signaling in human tumors may as such contribute to the observed efficacy of EGFR antagonists. Consistent with such an assumption is the fact that the best independent prognostic indicator for the efficacy of treatment in patients with tumors expressing a non-mutated form of K-Ras, is the expression level of AREG in the serum of cancer patients (Ishikawa et al., 2005; Ford et al., 2007; Jacobs et al., 2009; Tinhofer et al., 2011).

## Role of regulatory T cells in human cancer

In mouse tumor models the importance of Tregs has repeatedly been demonstrated. In contrast, their importance in established human tumors remains a more controversial and less well-studied subject. An elevated number of FoxP3-expressing CD4+ T cells have been described within several solid tumors; including ovarian and non-small-cell lung cancer tumors, and in the peripheral blood of breast, colorectal, and lung cancer patients (Zou, 2006). Data from patients suffering from a wide range of cancer types has further indicated that there appears to be a positive correlation between increased number of intra-tumoral FoxP3-expressing CD4+ T cells and poor prognosis for cancer patients (Curiel et al., 2004). Unfortunately, both Tregs and activated human CD4+ T cells transiently express the transcription factor FoxP3 (Wang et al., 2007). Therefore, an exact determination of the T cell phenotype of human FoxP3-expressing CD4+ T cells requires the analysis of the methylation status of the FoxP3 gene locus (Floess et al., 2007). Such analysis relies upon an assay only rarely performed on patient biopsy-derived T cells. In addition, the relevance of Tregs may be less important in the situation of established tumors that have already managed to circumvent and tolerize potential immune responses. Together this suggests that while Treg function may play a critical role at specific time points during the establishment of tumors, at the exact point at which the biopsy are taken Treg function may no longer play an essential role for tumor growth.

Nevertheless, clear evidence for the importance of Treg function during tumor immunotherapy exists. In recent years, the use of anti-CTLA-4 antibodies has gained significant support and success in the treatment of several types of tumors; supposedly, by reinvigorating tumor-specific cytotoxic CD8+ T cells suppressed by CTLA-4-expressing Tregs (Wolchok and Saenger, 2008). Furthermore, the importance of Tregs in limiting the efficacy of immunotherapy has been well-demonstrated in clinical cancer therapy. For example, it has been reported that the efficacy of vaccination against a high risk HPV type (HPV16) in patients with cervical cancer was directly correlated with the frequency of FoxP3-expressing CD25+ CD4+ T cells in patient peripheral blood following vaccination (Welters et al., 2010). Also, in clinical studies in which patients received chemotherapy followed by adoptive immunotherapy, the level of Treg reconstitution after transfer appeared inversely correlated with patient response to therapy (Yao et al., 2012). Thus, it is now assumed that the balance between effector T cells and Tregs may determine the impact of the anti-tumor therapy.

Collectively, these findings strongly suggest that Treg function might also play an important role in human tumor immune therapy, and we may assume that EGFR antagonist treatment-associated interference with Treg-mediated immune suppression enhances the efficacy of such cancer treatments.

## Improved applications of EGFR antagonists

**Figure 2:**
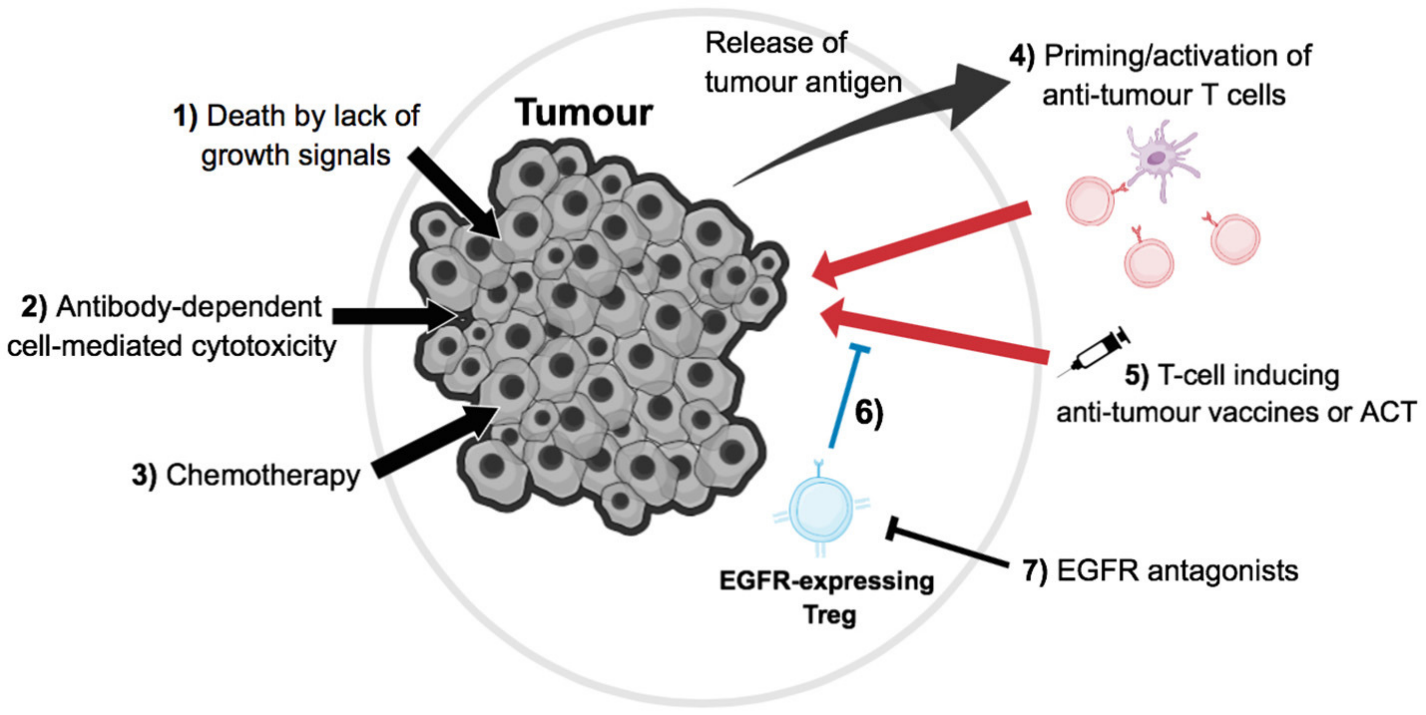
Direct and indirect effects of tumor immunotherapy. **(1)** For a subset of EGFR+ tumors, growth will be dependent on signals transmitted via the EGFR; therefore blocking this signal with EGFR antagonists could induce tumor cell death by lack of growth signals. **(2)** EGFR antagonists, such as monoclonal antibodies, can activate natural killer cell-mediated antibody-dependent cell death (ADCC) on EGFR-expressing cells, such as tumor cells and other immune cell populations in the microenvironment, thus ultimately leading to tumor cell death. **(3)** Chemotherapy is a common form of cancer treatment, which has a variety of effects such as tumor cell death and lymphopenia, which re-invigorates the anti-tumor T cell response. **(4)** Tumor cell death leads to the release of antigens, which can be presented by antigen presenting cells such as dendritic cells (purple) to prime or re-activate anti-tumor T cells (red). **(5)** Anti-tumor T cells can also be indirectly induced or directly transfused via vaccines or adoptive cell therapy respectively, which then can kill tumor cells. **(6)** These immune responses can be blocked by tumor-residential regulatory T cells (blue); however, **(7)** EGFR antagonists can hinder the suppression of Tregs that is induced via EGFR signaling, thus in turn aiding in tumor immunotherapy, as these Tregs no longer suppress anti-tumor T cells as efficiently.

The knowledge that EGFR inhibitor treatment may suppress the function of Tregs, thus aiding in improving the efficacy of tumor immunotherapy, allows the novel exploration of combination therapy. By combining current therapies with the use of EGFR antagonists, enhanced anti-tumor immune responses in cancer patients may be seen (Figure 2).

One such therapy includes tumor vaccination, which in the past has often failed in clinical applications, potentially due to a Treg-mediated immunosuppressive tumor microenvironment. It has already been demonstrated in a mouse model of B16 melanoma that combined therapy of tumor immunization with EGFR antagonist treatment improves the efficacy of either treatment alone (Zaiss et al., 2013). B16 tumors were transferred into *wt* C57BL/6 mice, and the mice later immunized with either tumor antigen-pulsed BM-DCs or EGFR-blocking nanobodies, BM-DCs in combination with EGFR-blocking nanobodies, or alone. Mice that were given nanobodies or antigen-pulsed BM-DCs alone showed little to no regression of tumor growth. However, mice that were immunized with BM-DCs in combination with nanobody treatment developed significantly smaller tumors; demonstrating the improved efficacy of combined therapies in this mouse model (Zaiss et al., 2013).

Another such treatment that could be combined with current EGFR inhibitor therapy is chemotherapy (CT). CT is currently one of the most common forms of cancer treatment, with the efficacy of CT depending on the type of cancer and the stage of tumor development. CT has a variety of effects on the immune system including, of importance, immunosuppression through depletion of immune cells such as dividing T cells. The group of Zitvogel and Kroemer have shown elegantly that CT can also induce and enhance anti-tumor immune responses in a number of ways (Zitvogel et al., 2008). Firstly, CT induces tumor cell death. As such, tumor (neo-) antigens are released and can subsequently be presented to tumor-specific T cells priming new immune responses against tumor cells or re-activating a dormant anti-tumor T cell response. In addition, CT-induced tumor cell death diminishes the overall tumor load temporarily, and thus with it the associated immunosuppressive microenvironment. However, most importantly for combined immunotherapy, CT transiently induces lymphopenia. This leads to substantial T cell proliferation that can again reactivate dormant T cell responses. It has further been well-established that in situations of lymphopenia, the activation of Treg populations plays an important role in dampening such dormant responses; for instance, to prevent the induction of autoimmune responses (Guerau-de-Arellano et al., 2009). The application of EGFR antagonists, and thus the suppression of Tregs function during this immune replenishment phase following CT-induced lymphopenia, may have the capacity to substantially improve the reactivation of anti-tumor immune responses and efficacy of CT (Figure 2). Of interest, in colon carcinoma patients, EGFR antagonist treatment appears efficient only in combination with chemotherapy.

Another promising line of combined therapy in cancer treatment is the combination of CT with adoptive cell transfer (ACT). In most cases, ACT describes the isolation of T cells from cancer patients. These T cells are then further cultured *in vitro*, and subsequently activated and expanded *ex vivo* with tumor-specific antigens prior to re-infusion. Alternatively, effector T cells are transduced with tumor-antigen specific TCRs eliciting a stronger anti-tumor immune response following later retransfer into the patient (June et al., 2015). ACT is normally combined with non-myeloid depleting CT prior to cell transfer, inducing lymphopenia-associated cell proliferation. One of the most critical aspects in determining the efficacy of ACT is thereby the rate in which Tregs expand following CT (Yao et al., 2012). Patients with a rapid recovery of Treg frequencies in the blood following therapy were mainly non-responders, while patients who had a delayed Treg recovery were responders (Yao et al., 2012). Therefore, it is tempting to speculate that the application of EGFR antagonists in combination with ACT during CT-induced lymphopenia, and the following recovery phase, could substantially diminish Treg function during a critical time point of treatment thus substantially improving the efficacy of such treatment (Figure 2).

## Outlook

Taken together, recent developments suggest a prominent immune involvement in the clinical efficacy of EGFR antagonist treatments. To fully verify such an involvement, it appears warranted that further and more focused analysis of tumor material—derived from cancer patients treated with EGFR antagonists—should be performed. These studies should address EGFR expression levels of intra-tumoral Treg populations during different stages of tumor development, and determine whether a shift in effector T cell to Treg ratio occurs in patients that undergo treatment with EGFR antagonists. Nevertheless, this newfound appreciation of the link between EGFR inhibitors and the immune system provides novel insights into how EGFR antagonists may function during cancer treatment, permiting adjustments to our current use of these drugs in the clinic. As the EGFR is expressed on a wide variety of cells, and is important for tissue homeostasis, off-target side effects of current EGFR inhibitors used in cancer treatment are common and can often be severe. To diminish such side effects, methods are required to be developed which can target EGFR antagonists specifically to tumor-residential Treg populations. As such, much lower concentrations of drugs would be needed as their effects would remain restricted to Tregs; thus enhancing the efficacy of treatment whilst diminishing side effects associated with EGFR antagonist treatment.

## Author contributions

FM and DZ, both discussed the outline of the review, the content, and the figures used. Both wrote the review and contributed to the drawing of the Figures.

### Conflict of interest statement

The authors declare that the research was conducted in the absence of any commercial or financial relationships that could be construed as a potential conflict of interest.
